# A novel RIP1/RIP3 dual inhibitor promoted OPC survival and myelination in a rat neonatal white matter injury model with hOPC graft

**DOI:** 10.1186/s13287-021-02532-1

**Published:** 2021-08-18

**Authors:** Chu Zhang, Qian Guan, Hao Shi, Lingsheng Cao, Jing Liu, Zixuan Gao, Wenxi Zhu, Yinxiang Yang, Zuo Luan, Ruiqin Yao

**Affiliations:** 1 Department of Cell Biology and Neurobiology, Xuzhou Key Laboratory of Neurobiology, Xuzhou Medical University, Xuzhou, 221004 People’s Republic of China; 2 Department of Histology and Embryology, Xuzhou Medical University, Xuzhou, 221004 People’s Republic of China; 3 Class ten, Grade two, Xuzhou Senior School, Xuzhou, 221003 People’s Republic of China; 4 Pediatrics, The Sixth Medical Center of PLA General Hospital, Beijing, 100048 People’s Republic of China

**Keywords:** Neonatal ischemia, hOPC graft, ZJU-37, White matter disease, Inflammasome

## Abstract

**Background:**

The dual inhibitors of receptor interacting protein kinase-1 and -3 (RIP1 and RIP3) play an important role in cell death processes and inflammatory responses. White matter injury (WMI), a leading cause of neurodevelopmental disabilities in preterm infants, which is characterized by extensive myelination disturbances and demyelination. Neuroinflammation, leads to the loss and differentiation-inhibition of oligodendrocyte precursor cells (OPCs), represents a major barrier to myelin repair. Whether the novel RIP1/RIP3 dual inhibitor ZJU-37 can promote transplanted OPCs derived from human neural stem cells (hOPCs) survival, differentiation and myelination remains unclear. In this study, we investigated the effect of ZJU-37 on myelination and neurobehavioral function in a neonatal rat WMI model induced by hypoxia and ischemia.

**Methods:**

In vivo, P3 rat pups were subjected to right common carotid artery ligation and hypoxia, and then treated with ZJU-37 or/and hOPCs, then OPCs apoptosis, myelination, glial cell and NLRP3 inflammasome activation together with cognitive outcome were evaluated at 12 weeks after transplantation. In vitro, the effect of ZJU-37 on NLRP3 inflammasome activation in astrocytes induced by oxygen–glucose deprivation (OGD) were examined by western blot and immunofluorescence. The effect of ZJU-37 on OPCs apoptosis induced by the conditioned medium from OGD-injured astrocytes (OGD-astrocyte-CM) was analyzed by flow cytometry and immunofluorescence.

**Results:**

ZJU-37 combined with hOPCs more effectively decreased OPC apoptosis, promoted myelination in the corpus callosum and improved behavioral function compared to ZJU-37 or hOPCs treatment. In addition, the activation of glial cells and NLRP3 inflammasome was reduced by ZJU-37 or/and hOPCs treatment in the neonatal rat WMI model. In vitro, it was also confirmed that ZJU-37 can suppress NLRP3 inflammasome activation in astrocytes induced by OGD. Not only that, the OGD-astrocyte-CM treated with ZJU-37 obviously attenuated OPC apoptosis and dysdifferentiation caused by the OGD-astrocyte-CM.

**Conclusions:**

The novel RIP1/RIP3 dual inhibitor ZJU-37 may promote OPC survival, differentiation and myelination by inhibiting NLRP3 inflammasome activation in a neonatal rat model of WMI with hOPC graft.

## Introduction

Preterm birth is a global public health issue [1]. According to estimates by the World Health Organization, there are nearly 15 million preterm births annually worldwide, with an overall incidence of 11.1% [2]. As many as 25–50% of preterm birth survivors develop chronic neurodevelopmental disorders, which manifest as cognitive, motor, and sensory disorders [3, 4], among which the white matter injury (WMI) is the most common [5]. It characterized by extensive myelination disturbances, demyelination and inflammatory reaction which can damage axons [6]. The period of highest risk for WMI is 23–32 weeks post-conception, during which time the pre-oligodendrocytes (pre-OLs) are more abundant than other cells [7] and the development of central nervous system (CNS) exhibits vulnerability to various insults such as hypoxia, ischemia, infection and inflammation [8]. Unfortunately, the pathogenesis of neonatal WMI is unclear and no specific therapies are currently available other than supportive treatment [9]. Studies thus far have suggested that remyelination is one of the pivotal mechanisms in promoting functional recovery following neonatal WMI [6, 10, 11] and that the inhibition of demyelination is one of the key challenges of therapeutic peptides.

As precursors of oligodendrocytes (OLs), oligodendrocyte precursor cells (OPCs) can proliferate and generate immature oligodendrocytes, which then differentiate into mature oligodendrocytes and wrap around axons to form the myelin sheath [12]. After inflammation-induced white matter damage, myelin regeneration occurs in demyelinating lesions, with endogenous OPCs maturing into myelin-producing oligodendrocytes; however, endogenous OPCs are in a state of relative insufficiency and gradually begin to failing to differentiate properly, and thus remyelination is incomplete [13]. Therefore, transplantation of exogenous OPCs may promote remyelination in the area of white matter lesions area [14]. Moreover, transplantation of embryonic stem cells (ESCs) [15], mesenchymal stem cells [16, 17], and neural stem cells (NSCs) [18] has been shown to enhance the repair of neurological deficits resulting from perinatal brain injury. Recently, OPC transplantation therapy has been investigated, including for lysophosphatidylcholine-induced demyelination [19], spinal cord injury [20–22], and radiation-induced injury [23], with therapeutic effects achieved in all animal models. Chen et al. [11] showed that transplanted mouse ESC-derived OPCs can migrate in vivo, differentiate into myelin, and ultimately provide neuroprotection to WMI mice. Given the species-specific differences in myelin development and regeneration between human and mouse OPCs [7], transplantation of OPCs derived from human neural stem cells (hOPCs) is clearly more conducive to clinical translational research. Moreover, our previous studies demonstrated that hOPCs alleviates demyelination, but are not abundant enough to completely repair this damage [24], so stimulating an endogenous regenerative response may be an effective treatment and many small-molecule compounds can promote OPC differentiation and/or remyelination.

Numerous studies have reported that neuroinflammation results in myelin-producing oligodendrocytes undergoing apoptosis and the loss of the myelin sheath. When the myelin sheath fails to regenerate, this ultimately leads to neurological disability [9]. Previous studies have reported astrocyte and microglial activation in demyelinating diseases. The diminishing of the inflammatory response during demyelination can contribute to nervous system recovery and prevent demyelination deterioration [25, 26]. Inflammasomes are a family of cytosolic pattern recognition receptors (PRRs) [27]. The Nod-like receptor pyrin domain containing 3 (NLRP3) recruits and activates pro-caspase-1 into caspase-1 through ASC (apoptosis associated speck-like protein containing caspase recruitment domain) and then forms the inflammasome, which leads to the release of IL-1β and IL-18 into the extracellular environment and induces neuroinflammation and damage to axons [25, 28, 29]. It was recently verified that the NLRP3 inflammasome is overactivated during demyelinating disorders [26]. Our previous studies demonstrated that inhibition of NLRP3 inflammasome activation can alleviate demyelination in the corpus callosum induced by cuprizone [9, 25, 30]. Therefore, molecules that regulate NLRP3 may prevent demyelination and restore neurological dysfunction of neonatal WMI by reducing inflammatory reactions.

Receptor interacting protein kinase-1 and -3 (RIP1 and RIP3) are involved in cell death and inflammatory responses [31]. An inhibitor of RIP1, necrostatin-1 (Nec-1), as a therapeutic drug for neurological diseases, can effectively inhibit programmed necrosis, RIP1-dependent apoptosis and various inflammatory responses [32]. Nec1 has been clinically confirmed to regulated inflammation in microglial and apoptosis in oligodendrocytes in a recent study, suggesting its therapeutic benefit for demyelination in vitro and in vivo [33, 34]. RIP3 can promote RIP1 phosphorylation and then form a stable phosphorylated RIP1 and RIP3 complex with a significant increase in intracellular reactive oxygen species [35]. Inhibitor of RIP3 showed more potent inhibitory activity against inflammation than inhibitor of RIP1, but have not entered the clinic for patients because of cell apoptosis [35]. The research group of Professor Hongguang Xia of Zhejiang University designed and synthesized chemical drug ZJU-37 which is a novel class of inhibitors with dual targeting ability to both RIP1 and RIP3 and a China National Invention Patent (ZL201810826696.1, China) has been awarded. ZJU-37 has been shown to play an important role in cell death processes and inhibit inflammatory responses but does not induce apoptosis in a variety of animal and cell models. ZJU-37 also has therapeutic potential for the neurodegenerative diseases, autoimmune diseases, gastroenteritis and acute liver injury. Whether ZJU-37 can participate in demyelination in the neonatal rat WMI model remains unclear, and relatively few studies have focused on the effects of ZJU-37 on the injury and functional recovery of OPCs.

To determine whether ZJU-37 promotes remyelination more efficiently with hOPC graft, we transplanted hOPCs into WMI rat brains with ZJU-37 intraperitoneal injection, and investigated the myelination and neurobehavioral functions. Furthermore, we intended to provide novel insights into the role of ZJU-37 in the survival, differentiation and myelination of hOPCs in vitro and explored the related mechanism. The data presented here demonstrate for the first time that ZJU-37 not only promotes OPC survival, differentiation, and myelination by inhibiting NLRP3 inflammasome activation in a neonatal rat WMI model with hOPC graft, but also promoted myelination and improved behavioral function with hOPC graft compared to ZJU-37 or hOPCs alone.

## Materials and methods

### Preparation and identification of hOPCs

HOPCs derived from NSC spheres which were generated from the CNS tissue of spontaneously aborted human female fetuses at gestational week 10–12 from the Pediatrics Laboratory of the Sixth Medical Center of PLA General Hospital (Beijing, China) and the experimentation protocol was approved by the institutional ethics committee (Application No. 2015013). Informed written consent was obtained from the mothers, and NSCs differentiated into hOPCs which has good proliferation ability and can be stably expanded to the fifth generation in vitro according to a modified version of a previously published protocol [36]. Briefly, the brain was homogenized into a suspension of single cells by mechanical dissociation. Cells were trypsinized and transferred to a neural differentiation medium containing DMEM/F12, leukemia inhibitory factor, basic fibroblast growth factor, epidermal growth factor, and nonessential amino acids (all from Gibco, Waltham) and hOPCs were cultured in OPC medium as previously described [36]. Viability of transplanted hOPCs was confirmed by cell morphology and immunofluorescence staining of specific markers ST8 alpha-N-acetyl-neuraminide alpha-2,8-sialyltransferase 1 (A2B5, rabbit IgG, 1:200, Abcam), platelet derived growth factor receptor alpha (PDGFRα, rabbit IgG, 1:800, Cell Signaling Technology), chondroitin sulfate proteoglycan 4 (NG2, rabbit IgG, 1:200, Millipore), oligodendrocyte marker O4 (O4, mouse IgG, 1:200, Sigma) and glial fibrillary acidic protein (GFAP, mouse IgG, 1:500, Santa Cruz).

### Neonatal rat WMI model and treatment

Clean-grade SD rat pups (P3) obtained from the Center of Experimental Animals of Xuzhou Medical University were randomly divided into control (Ctrl), WMI and vehicle-treated (Vehicle) group, WMI with ZJU-37-treated (ZJU-37) group, WMI with hOPCs transplantation (hOPCs) group and WMI with ZJU-37-treated and hOPCs transplantation (ZJU-37 + hOPCs) group. Animals were housed in an air-conditioned room with a 12 h light/dark cycle, and provided with adequate food and water. All efforts to reduce the number of animals used and minimize animal suffering were made. All WMI groups were generated as described previously [36]. Briefly, P3 pups were placed in a refrigerator at − 20 °C for 7–10 min. After freezing anesthesia, the right common carotid artery was carefully isolated from the surrounding tissue and ligated. Then, the wound was sutured with an 8-0 suture and the time of operation was controlled to within 5 min. Upon completion of the surgery, pups were moved to the recovery area under a heat lamp for 10 min, returned to their mother, and allowed to recover for an additional 1 h before exposure to 6% oxygen (94% nitrogen saturation) at 37 °C for 90 min in a humidified chamber. After monitoring recovery, the pups were returned to cages to continue feeding. Sham-operated rats without hypoxia were used as the Ctrl group. At P4, P6, P8 and P10, the degree of injury (mild/moderate to severe) was determined by observing the general condition of animals, neurobehavioral evaluation, and histopathology. Moderately to severely injury rats were used for subsequent experiments.

For the immunosuppression, all transplant recipients received cyclosporine (i.p, 10 mg/kg) daily three days before transplantation and continuing for a total of four weeks. Subsequently, the rats were administered cyclosporine (100 μg/mL) via their drinking water until perfusion. During the immunosuppression period, the cyclosporine dose was appropriately reduced or discontinued if the rats were found to have sustained weight loss, red eyelids, or nasal damage.

At P12, the ZJU-37 group was injected intraperitoneally with ZJU-37 (i.p, 10 mg/kg) once daily for the first month, every other day for the second month and every third day for the third month until euthanasia. The Vehicle group was administered DMSO (i.p, 10 mg/kg) whereas rats in the Ctrl group received no treatment. Rats in the cell transplantation groups were fixed on a stereotaxic apparatus after narcotization with 4% chloral hydrate (4 mL/kg) at P12. A small incision was made through the midline to expose the skull. The anterior fontanelle was used as a guide to determine the puncture point whose coordinates from bregma were as follows: anteroposterior-1.0 mm; mediolateral-1.5 mm; dorsoventral-2.0 mm by brain stereotaxic apparatus. A 5 μL microsyringe was used to withdraw 3 μL cell suspension (approximately 3 × 10^5^ hOPCs), which was then slowly injected into the transplant site. After injection, the needle was left at the injected site for an additional 5 min then slowly withdrawn. Subsequently, the scalp wound was closed and the rats were placed back into home cages and nursed after fully awaking from anesthesia under a heat lamp. The transplantation time for each rat was approximately 30 min. In the ZJU-37 + hOPCs group, ZJU-37(i.p, 10 mg/mL) was administered daily for the first month after transplantation, every other day for the second month, and every third day for the third month until euthanasia. In the hOPCs group, DMSO was administered instead of ZJU-37.

### Behavioral tests

All behavioral experiments were performed during the 11 weeks after transplantation of eight rats each group.

### Morris water maze (MWM) test

This test was performed as previously described [37]. Briefly, each rat entered the water training from four different quadrants and underwent two training sessions per day for five consecutive days. The latency to escape the water maze (the time from the rats entering the water to standing on the platform) was counted for each trial. On day 6, a probe test was performed by removing the platform and allowing each rat to swim freely for 60 s after entering the water from the first quadrant. The number of platform crossings and number and time that rats crossed through the platform quadrant were recorded. All data were recorded with a computerized video system.

### Limb-use asymmetry test (cylinder)

The forelimb-use asymmetry test was used to assess sensorimotor function and behavioral symmetry. Rats were placed in a transparent Plexiglas cylinder (40 cm high, 20 cm diameter) [38] and initial forepaw (left/right/both) preference for weight-bearing contacts was scored. The forelimb asymmetryscore was calculated as: (right-left)/total of number of contacts.

### Adhesive removal test

Sensory and motor functions were measured as described previously [39]. All rats were familiarized with the testing environment for three days in a Perspex box. Two adhesive tapes were placed with equal pressu recovering the hairless parts on both forelimbs. The time to remove the adhesive tapes from each forelimb was recorded within a maximum of 120 s.

### Histological examination

Rats from each group were deeply anesthetized with chloral hydrate at 12 weeks after transplantation and intracardially perfused with Phosphate-buffered saline (PBS) followed by fixation with 4% cold paraformaldehyde. Brains were dissected and post-fixed in the same solution for 12 h at 4 °C, then sequentially dehydrated in sucrose (15% and 30%) until permeated. Coronal sections (14 µm thickenss) were cut on a freezing microtome (Leica Microsystems, Nussloch, Journal Pre-proof Germany) from the bregma anterior–posterior coordinates + 1.0 to − 1.0, collected on 3-aminopropyltriethoxysilane-coated slides (Sigma, St. Louis, MO, USA), and stored at − 80 °C in cryoprotectant solution. For protein analyses, fresh corpus callosum from sacrificed rats (4 from each sub-group) were isolated and stored at − 80 °C.

### TUNEL staining

OPC apoptosis was assessed by TUNEL staining via a Dead End Fluorometric TUNEL System (Roche, Switzerland). Brain tissues were incubated overnight at 37 °C with the anti-PDGFRα (1:800) antibody. A standard TUNEL procedure was performed as described previously [9] after probing with a relevant secondary antibody. Nuclei were stained using 4′,6-diamidino-2-phenylindole (DAPI, Beyotime Biotechnology, Shanghai, China). TUNEL-positive cells were counted at five randomly chosen microscopic fields. OPC apoptotic rate was calculated as TUNEL and PDGFRα double-positive cells/total PDGFRα-positive cells per field × 100%.

### Immunohistochemistry

Immunofluorescence staining was performed on the above-described sections of brain tissue (20 µm). Sections were blocked for 1 h in PBS containing 5% bovine serum albumin and 0.3% Triton X-100 at room temperature then incubated overnight with primary antibody. The following primary antibodies were used: anti-STEM121 (IgG, 1:500, TaKaRa), anti-myelin basic protein (MBP, IgG, 1:1000, Abcam), anti-glial fibrillary acidic protein antibody (GFAP, IgG, 1:500, Santa Cruz), anti-ionized calcium binding adaptor molecule-1 (Iba-1, IgG, 1:1000, Wako) and anti-NLRP3 (IgG, 1:500, Novus Biologicals). After washing three times with PBS, the samples were incubated with goat anti-mouse or goat anti-fluorescein isothiocyanate (FITC, IgG, 1:200, Santa Cruz) for 2 h at room temperature. Nuclei were stained with DAPI according to the manufacturer's instructions. Staining specificity was assessed by omitting the primary antibody. The number of GFAP-positive and Iba-1-positive cells was counted at three sections per rat from the same levels. Quantitative analysis of immunofluorescence staining was measured by Image J software (NIH, Bethesda, MD, USA).

### Transmission electron microscopy (TEM)

Samples were prepared for electron microscopy according to previous protocols [40]. In brief, brains were removed quickly after perfusion with 2% PFA/2.5% glutaraldehyde. The corpus callosum corresponding to the transplantation site (*n* = 4 per group) were dissected and placed in 2.5% glutaraldehyde at 4 °C overnight for post-fixation. After transferring to osmium tetroxide for 1 h at room temperature and dehydrating with increasing ethanol concentrations, the tissues were embedded in epoxy resin embedding medium. Ultrathin sections (50 nm) were made from the resin-embedded samples and observed under a transmission electron microscope. A total of 100 root axonal fibers from three samples in each group were measured at a magnification of 15,000×*g*. Following image acquisition, axon and myelin diameters were measured using Image J software. The g-ratio, which is a structural index of remyelination and defined as the ratio of the inner axonal diameter to the total outer fiber diameter and lower ratios indicate more extensive myelination, was subsequently assessed. The average scores of ultrastructural myelin damage were determined as described previously [9].

### Primary cell culture and drug treatment

Extraction and culturing of primary rat astrocytes and OPCs from newborn 0–2 day-old rat cerebral cortices were performed as previously described [41]. Digestions were stopped with DMEM/F12 (1:1, HyClone) containing 10% fetal bovine serum (FBS, Clark Bioscience, Richmond). Cells were centrifuged at 1200×*g* for 5 min, and supernatant was discarded. The medium was changed once every 2–3 days. After 9–11 days, microglia were dislodged using an orbital shaker (200×*g* for 1 h, 37 °C), and OPCs were harvested by collecting the cell suspension after shaking the on ahorizontal orbital shaker for 18 h at 200×*g* and 37 °C. The cells were then digested with 0.25% trypsin and seeded into 24-well plates at an appropriate density; the remaining adherent cells were astrocytes. Cells after the third generation were used for experiments and were divided into normal (Ctrl) and oxygen and glucose deprivation (OGD) groups. OGD-treated astrocytes were further divided into OGD, OGD-ZJU-37(5 µM) and OGD-ZJU-37(10 µM) groups. The cells were treated with ZJU-37 for 30 min and then subjected to 6 h of hypoxia in an incubator containing 1% oxygen and 95% nitrogen and then reoxygenated for 24 h. Finally, cells were collected for cellular immunofluorescence staining and western blot analysis. The astrocyte supernatant was mixed with DMEM/F12 at a ratio of 1:1. After OPCs grew for 2 days in DMEM/F12 containing 10% FBS, the mixed medium without OGD-injured astrocytes was used for the control (Ctrl) group and the conditioned medium from OGD-injured astrocytes (OGD-astrocyte-CM) with DMSO or ZJU-37 were used for the CM-vehicle group, the CM-ZJU37 (5 μM) group and the CM-ZJU37 (10 μM) group respectively for 24 h. Finally, the OPCs were collected for cellular immunofluorescence staining of PDGFRα and MBP and the Annexin V-FITC/PI to detect apoptosis by flow cytometry, separately.

### Western blot analysis

Western blotting was performed as previously described [9]. Total protein was extracted from the corpus callosum of the WMI rats and primary rat astrocytes were lysed with a lysis buffer and homogenized and centrifuged at 12,000×*g* for 15 min at 4 °C. The supernatants were collected and used for protein detection. Samples were run on a 10% SDS-PAGE gel and transferred tonitrocellulose membranes. Primary antibodies were: anti-NLRP3 (IgG, 1:500, Novus Biologicals), anti-ASC (IgG, 1:500, Santa Cruz), anti-caspase-1 (IgG, 1:500, Santa Cruz), anti-cleaved caspase-1 p20 (IgG, 1:1000, Cell Signaling Technology) and anti β-actin (IgG, 1:1000, Santa Cruz). The gray value of every band was analyzed with Image J software and reported as relative optical density of the specific proteins.

### Statistical analysis

Three independent replicates were conducted for each experiment. Experimental data were analyzed with GraphPad Prism® software. After variation similarity was compared, one-way analysis of variation (ANOVA) followed by either the Newman-Keuls or Tukey honestly significant difference post hoc test was used for comparisons among multiple groups. A two-way ANOVA was used for escape latency in the Morris water maze training task. Quantitative data are expressed as mean ± standard error of mean. Statistical significance was set at *p* < 0.05 for all tests.

## Results

### Transplanted hOPCs produce myelin sheath

**Fig. 1:**
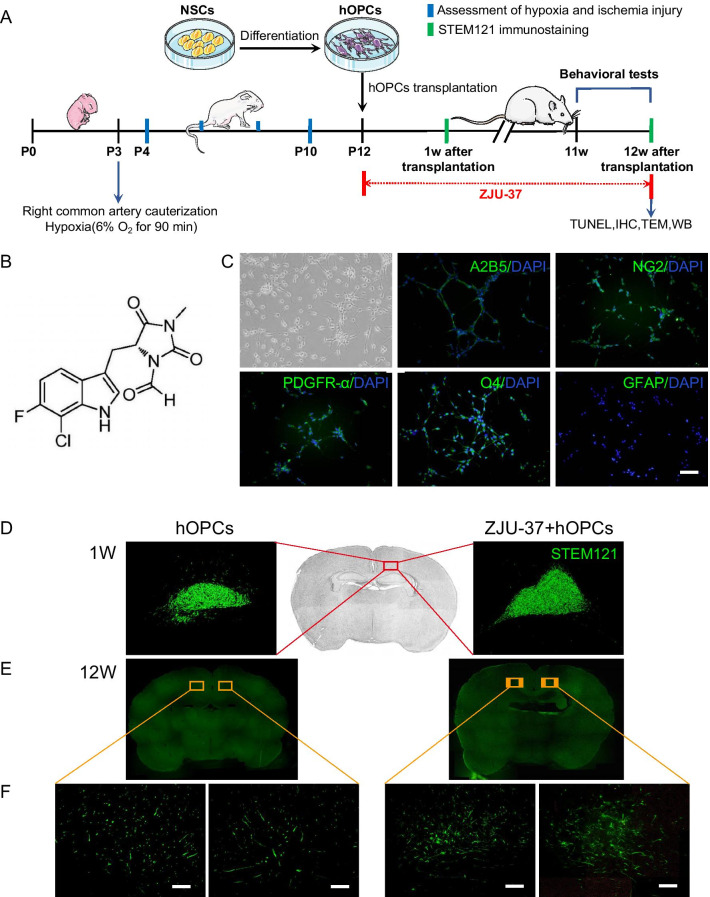
Morphology, migration and distribution of hOPCs. **A** Schematic illustration of the experimental manipulation. P3 rat pups were used to prepare the hypoxic-ischemic model; hOPCs were injected into the transplant site and ZJU-37 was intraperitoneally injected at P12; rats were sacrificed immediately after the Morris water maze (MWM) test. **B** The chemical structure of ZJU-37.** C** Identification of the bright field morphology and immunofluorescence staining to detect the NPC or OPC marker A2B5 (green), the pre-oligodendrocyte marker NG2 (green), the OPC marker PDGFRα (green), the immature oligodendrocyte marker O4 (green) and the astrocyte marker GFAP (green) of transplanted hOPCs. Scale bar = 100 μm. **D-E** Transplanted cells stained with human-specific marker STEM121 (green) at 1 week and 12 weeks post-transplant in the hOPCs and ZJU-37 + hOPCs groups. **F** At higher magnification of the boxed area from the STEM121 + cells exhibited typical bipolar OPC morphology. Scale bar = 100 μm. n = 4 for each group

The hOPCs showed a bipolar or multipolar morphology under phase-contrast microscopy and expressed the NPC or OPC marker A2B5, the pre-oligodendrocyte marker NG2, the OPC marker PDGFRα and the immature oligodendrocytes marker O4 fluorescence (Fig. 1C), which were able to be stably passaged up to the fifth generation. No astrocyte marker GFAP-positive cells were found in vitro (Fig. 1C). These results confirmed the characteristics of harvested hOPCs. To determine whether ZJU-37 could promote a more effective engraftment in vivo, hOPCs were injected into the corpus callosum of WMI rats. We stained brain tissue with human-specific marker STEM121 to confirm that the transplanted cells were human-derived. The retention of hOPCs was indicated by the presence of TEM121-positive cells at 1 and 12 weeks after transplantation at the injection site. At 1 week after transplantation, grafted cells revealed by STEM121 were found near the transplant site in all transplanted animals (Fig. 1D). The animals without cell transplantation did not exhibit such labelling. At 12 weeks after transplantation, STEM121-positive cells were detected in the contra lateral and anteroposterior directions, and were also observed migrating along the white matter tract of the corpus callosum (Fig. 1E), confirming that transplanted hOPCs can penetrate the BBB and migrate into the host brain. More STEM121-positive cells were observed in the ZJU-37 group, and both groups exhibited long-term survival. At higher magnification, the STEM121-positive cells adopted a typical bipolar branched OPC morphology (Fig. 1F). Teratomas, tumours, and non-neuronal tissue formation were not observed in the transplant recipients over the course of the experiment.

**Fig. 2:**
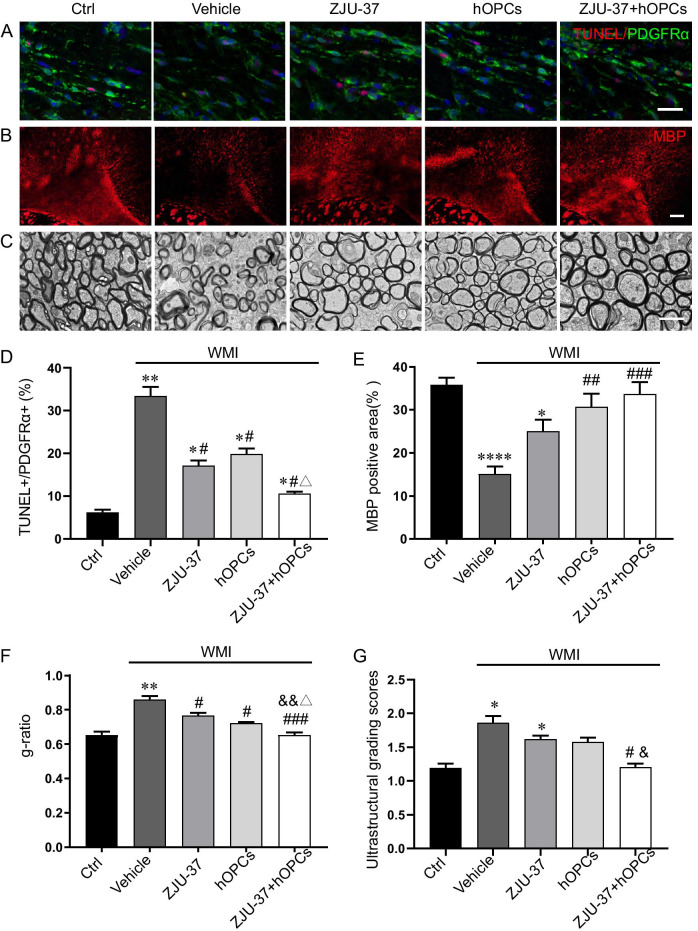
ZJU-37 combined with hOPCs effectively promotes OPC survival and myelination in WMI rats. **A** HI-induced OPC apoptosis was detected by PDGFRα (green) immunofluorescence staining and TUNEL staining (red) in the corpus callosum of each group, scale bar = 20 μm (**A**). The percent of TUNEL and PDGFRα double-positive cells was analyzed (**D**). **B** Myelination ability of ZJU-37 and transplanted hOPCs were assessed by myelin basic protein MBP (red) immunofluorescence staining (**B**) and quantitative analysis (**E**). Scale bar = 50 μm. **C** Representative electron microscopic images of the the myelin sheath in the corpus callosum were shown of each group. Magnification: 20,000×. **F** The g-ratio was calculated in each group. **G** Ultrastructural myelin-damage score was calculated in each group. n = 4 for each group. Data are presented as the mean ± SEM. *P < 0.05, **P < 0.01, ****P < 0.0001 vs. Ctrl group; ^#^*P* < 0.05, ^##^*P* < 0.01, ^###^*P* < 0.001 vs. Vehicle group; ^&^*P* < 0.05, ^&&^*P* < 0.01 vs. ZJU-37 group; ^△^*P* < 0.05 vs. hOPCs group

MBP, a structural protein with an indispensable role in myelin thickening and compaction, was observed to detect the differentiation of OPCs and the status of myelin by immunofluorescence staining at 12 weeks after transplantation. The remyelination fluorescence signal was observed in all WMI groups, and the bundles of MBP-positive nerve fibers were denser in the ZJU-37 + hOPCs group than the other groups (Fig. 2B, E). The percentage of the MBP-positive area in the Vehicle group was significantly lower than in the Ctrl group. Additionally, compared with the Vehicle group, the percentages of the MBP-positive area in the three treatment groups were significantly increased to different degrees, and which were more pronounced in the two transplantation groups (Fig. 2E). The results suggest that transplanted hOPCs can perform myelination and ZJU-37 effectively promote OPC differentiation in vivo.

### ZJU-37 combined with hOPCs more effectively decreased OPC apoptosis, and promoted remyelination of WMI rats compared to ZJU-37 or hOPCs treatment alone

OPC apoptosis of the neonatal rat WMI model was detected by TUNEL and PDGFRα immunofluorescence staining, which confirmed that there was a clear increase in the percent of TUNEL and PDGFRα double-positive cells in the Vehicle group. The three treatment groups showed marked reductions in OPC apoptosis to varying degrees, with the ZJU-37 + hOPCs group showing the lowest percent compared to the Vehicle group (Fig. 2A, D). Based on the results obtained for MBP immunofluorescence staining, ZJU-37 appeared to not only suppress the apoptosis of OPCs, but also promote the differentiation of OPCs and the maturation of oligodendrocytes in WMI rats. Under transmission electron microscopy, the structure of the myelin sheath in the Ctrl group maintained its integrity. The Vehicle group exhibited significant ultrastructural alterations in the myelin and axons, such as reduced thickness of the myelin sheath, a clearly disordered and loosened of myelin lamellar arrangement, and an irregular and sporadic arrangement of myelin sheath regeneration around the axons. In contrast, evidence of structural repair was observed in demyelinated areas in the three treatment groups, with some of the myelin sheath appearing normal in shape and at an increased abundance compared to the Vehicle group (Fig. 2C). However, some areas of myelin sheath still exhibited an abnormal structure, including insufficient myelin formation, division, and vacuolation. The g-ratio increased markedly in the Vehicle group compared to the Ctrl group. More thicker and compact myelin sheaths and less disturbed myelin sheaths were found in the ZJU-37 + hOPCs group (Fig. 2C). The three treatments led to a significant decrease in the g-ratio. Additionally, the ZJU-37 + hOPCs group exhibited a more compact and thicker myelin structure compared to the ZJU-37 and the hOPCs groups (Fig. 2E). The average score of ultrastructural myelin damage was significantly higher in the Vehicle group and was significantly attenuated in the ZJU-37 + hOPCs group, which nearly returned to the Ctrl group (Fig. 2F). These data indicate that ZJU-37 combined with hOPCs transplantation promoted remyelination more efficiently than ZJU-37 or hOPC transplantation alone.

### ZJU-37 combined with hOPCs more effectively improved cognitive and motor function of WMI rats compared to ZJU-37 or hOPCs treatment alone

**Fig. 3:**
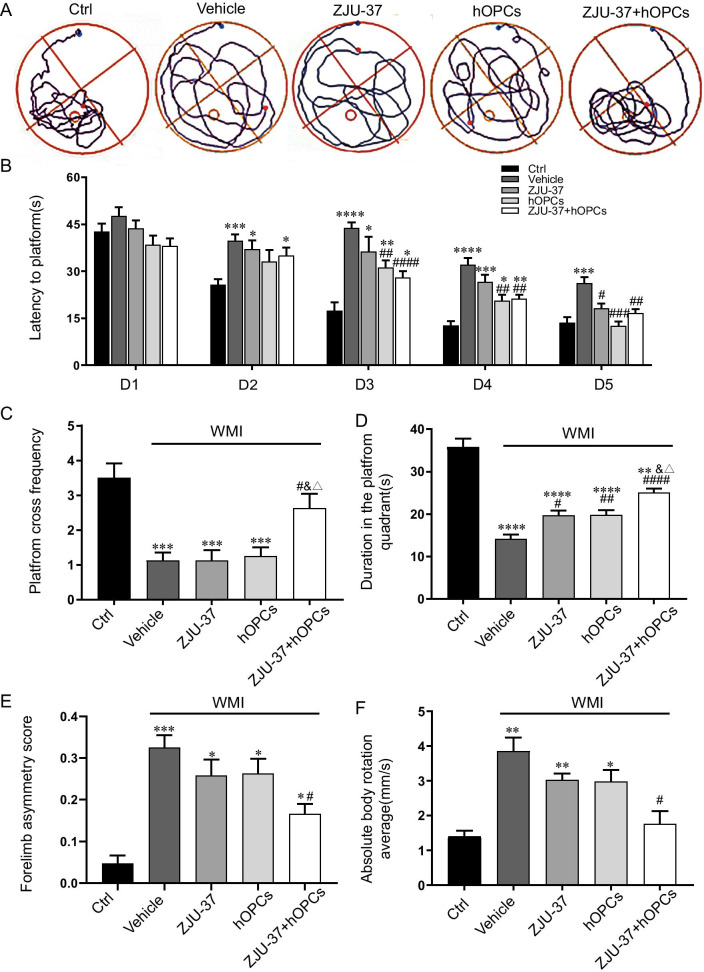
ZJU-37 combined with hOPCs improves behavioral function in WMI rats. **A** Representative tracking from each group in the MWM test on day 6 (small circles, location of the platform; blue and red points, start and end locations of the rats, respectively). **B** Average latency to find a hidden platform during the first 5 days of training in the directional navigation experiment for each group. **C** Number of platform crossings in the spatial exploration experiment on day 6 when the platform was removed. **D** The time spent in the target quadrant on day 6 of the MWM. **E** Forelimb asymmetry score in cylinder test of each group. **F** Mean time to remove the patch in the adhesive removal test of each group. *n* = 8 for each group. Data are presented as the mean ± SEM. *P < 0.05, **P < 0.01, ***P < 0.001, ****P < 0.0001 vs. Ctrl group; ^#^*P* < 0.05, ^##^*P* < 0.01, ^####^*P* < 0.0001 vs. vehicle group; ^&^*P* < 0.05 vs. ZJU-37 group; ^△^*P* < 0.05 vs.hOPCs group

To determine whether ZJU-37 combined with hOPCs transplantation could recover long-term neurological damage due to WMI, we performed behaviour analysis. Morris water maze tests were performed on rats for five consecutive days to evaluate place navigation. The results showed that all of the rats had same performance at Day 1. The Vehicle group rats had longer escape latencies than the Ctrl group from Days 2. The three kinds of treatments decreased the escape latency of WMI rats from Day 3 to Day 5 in the acquisition/learning phase (Fig. 3B), whereas the ZJU-37 + hOPCs group and the Ctrl group had no significant differences at Day 5. On the sixth testing day, the platform was removed for the probe test, and the number of times that the rats crossed the target region was significantly increased in the ZJU-37 + hOPCs group compared to the Vehicle group (Fig. 3C) and the time spent in the target quadrant was markedly longer in the three treatment groups than in the Vehicle group. Furthermore, this time period was longer in the ZJU-37 + hOPCs group than ZJU-37 and hOPCs groups (Fig. 3D). These results indicate that ZJU-37 combined with hOPCs treatment significantly reversed learning ability and reference memory function.

The cylinder test showed that forelimb movement in the control group was symmetrical, that WMI exacerbated forelimb-use asymmetry in the animals, and ZJU-37 combined with hOPCs transplantation prevented advancements in forelimb-use asymmetry but not to normal levels (Fig. 3E). The adhesive removal test, which is a sensitive approach used to evaluate sensorimotor deficits, revealed functional deficits in the Vehicle group; ZJU-37 combined with hOPCs transplantation reduced the mean time to remove the patch (Fig. 3F). Based on these results, ZJU-37 combined with hOPCs transplantation significantly ameliorated neurological deficits and improved somatosensory functions in the WMI rats.

### The activation of glial cells and NLRP3 inflammasome was reduced by ZJU-37 or/and hOPCs treatment in a neonatal rat WMI model

**Fig. 4:**
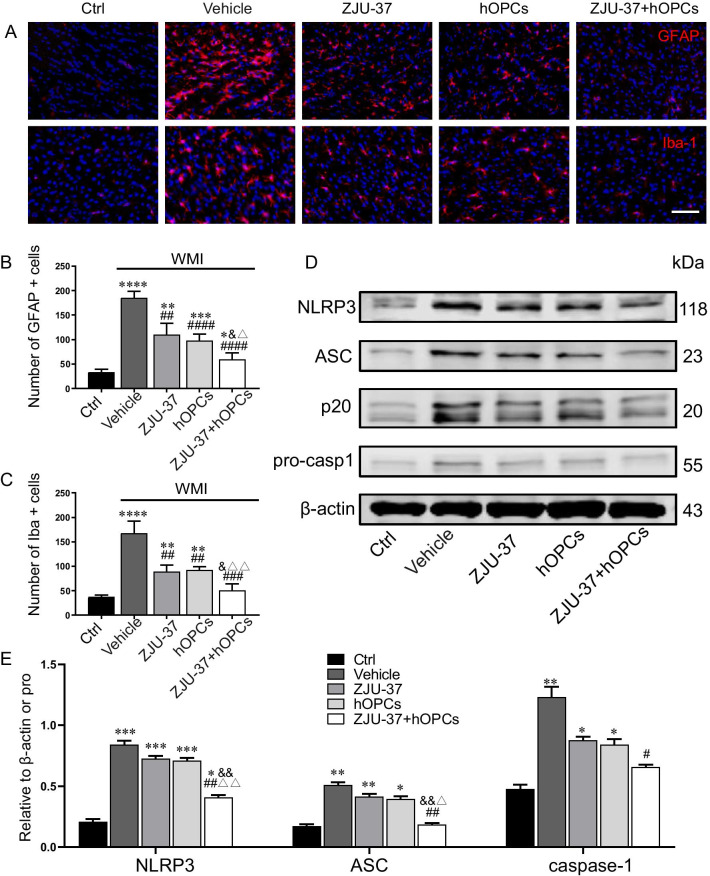
ZJU-37 or/and OPCs treatment reduces neuroglial and NLRP3 inflammasome activation in WMI rats. **A** The activation of glial cells was detected by GFAP (red) and Iba-1 (red) immunofluorescence staining in the corpus callosum of each group. Scale bar = 100 µm. **B-C** quantitative analysis of the GFAP + and Iba-1 + cells number of each group. **D-E** Western blotting and quantification for the expression of NLRP3, ASC, and cleaved caspase-1 (p20) in the corpus callosum for all groups. *n* = 4, triplicates per group. Data are presented as the mean ± SEM. *P < 0.05, **P < 0.01, ***P < 0.001, ****P < 0.0001 vs. Ctrl group; ^#^*P* < 0.05, ^##^*P* < 0.01, ^###^*P* < 0.001, ^####^*P* < 0.0001 vs. Vehicle group; ^&^*P* < 0.05, ^&&^*P* < 0.01 vs. ZJU-37 group; ^△^*P* < 0.05, ^△△^*P* < 0.01 vs. hOPCs group

It has been reported that astrocyte, microglia and the NLRP3 inflammasome are activated in demyelination animal models [25, 26]. Immunofluorescence staining was used to determine whether ZJU-37 alleviated demyelination by inhibiting glial activation. As shown in Fig. 4A, increased numbers of positive cells for GFAP (a marker of astrocytes) and Iba-1 (a marker of microglia cells) were observed in the corpus callosum of the Vehicle group, demonstrating that astrocytes and microglias were significantly over-activated, but ZJU-37 or/and hOPCs treatment significantly decreased the number, with the ZJU-37 + hOPCs group close to the Ctrl group (Fig. 4B, C). Western blotting showed that NLRP3, ASC, and cleaved caspase-1 (p20) protein expression was significantly increased in WMI rats, whereas treatment with ZJU-37 combined with hOPCs clearly reduced this level (Fig. 4D, E). This suggests that the NLRP3 inflammasome was activated in the neonatal rat WMI model and ZJU-37 may alleviate demyelination by inhibiting activation of the NLRP3 signalling pathway.

### ZJU-37 suppresses NLRP3 inflammasome activation in astrocytes induced by oxygen–glucose deprivation (OGD)

**Fig. 5:**
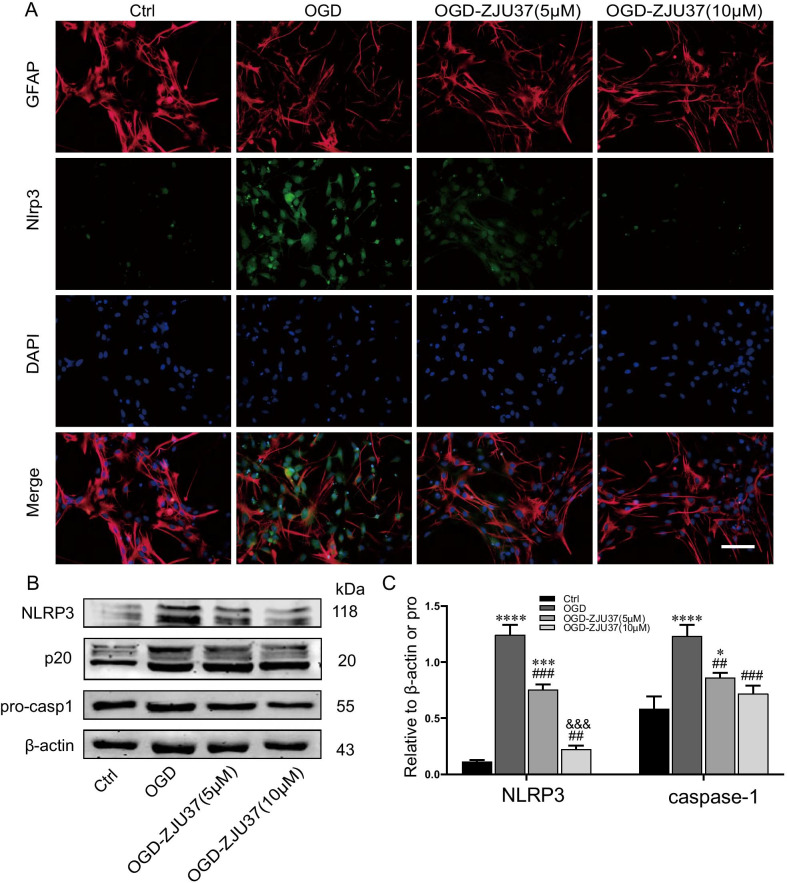
ZJU-37 suppresses NLRP3 inflammasome activation in astrocytes induced by OGD in vitro. **A** Representative images of GFAP (red) and NLRP3 (green) immunofluorescence staining in each group of cells cultured in DMEM/F12 medium containing 10% FBS without ZJU-37 and reoxygenated for 24 h of each group. Scale bar = 100 µm. **B-C** Western blots and quantitative analyses of NLRP3 protein and cleaved caspase-1 (p20) protein expression in primary cultured rat astrocytes of each group. *n* = 4, triplicates per group. Data are presented as the mean ± SEM. *P < 0.05, ***P < 0.001, ****P < 0.0001 vs. Ctrl group; ^##^*P* < 0.01, ^###^*P* < 0.001 vs. OGD group; ^&&&^*P* < 0.001 vs. OGD-ZJU37 (5 μM)

Immunofluorescence analysis revealed intense immunoreactivity of NLRP3 in OGD (6 h)-treated primary cultured astrocytes (Fig. 5). Concomitant incubation with ZJU-37 significantly attenuated the OGD-induced immunofluorescence activities and expression of NLRP3 with more pronounced in the 10 μM group than in the 5 μM group (Fig. 5A–C). Moreover, ZJU-37 reduced OGD-elevated p20 expression (Fig. 5B, C). These data suggest that ZJU-37 can inhibit OGD-induced astrocyte NLRP3 inflammasome activation in vitro.

### ZJU-37 attenuated OPC apoptosis and dysdifferentiation caused by the OGD-astrocyte-CM

**Fig. 6:**
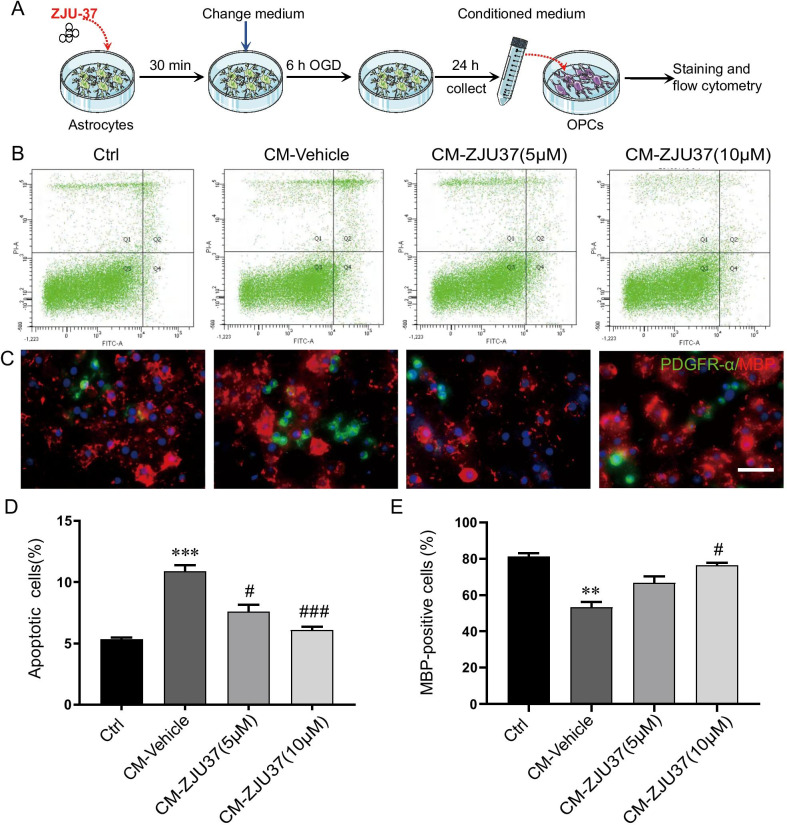
ZJU-37 attenuats OPC apoptosis and dysdifferentiation caused by the OGD-astrocyte-CM. **A** Flow chart of the experiment with conditioned medium transfer from OGD-injured astrocytes to OPCs in culture. Mixed medium of OGD astrocyte supernatant and DMEM/F12 was used for OPCs group for 24 h. **B** Annexin V-FITC and PI staining was used to detect apoptosis of primary cultured rat OPCs after incubated for 24 h with the OGD-astrocyte-CM. **C** Representative immunofluorescence staining of PDGFR-α (green) and MBP (red) counterstained with DAPI (blue) of each group. Scale bar = 100 µm. **D** Quantitative analysis of apoptosis rate of OPCs of each group. **E** Quantitative analysis of the percentage of MBP-positive cells of each group. *n* = 4, triplicates per group. Data are presented as the mean ± SEM. **P < 0.01 vs. Ctrl group; ^#^*P* < 0.05 vs. CM-Vehicle group

We used the OGD-astrocyte-CM treated with ZJU-37 to incubate OPCs and then detected the apoptosis and differentiation of OPCs. The percentage of apoptotic OPCs was markedly increased in the CM-Vehicle group compared to the Ctrl group. ZJU-37 reversed the increase in the apoptotic rate of OPCs induced by the OGD-astrocyte-CM, with the 10 µM group showing more effects (Fig. 6B, D). The immunofluorescence double staining showed that more PDGFRα-positive cells and less MBP-positive cells were observed in the CM-Vehicle group while less PDGFRα-positive cells and more MBP-positive cells were observed in the ZJU-37 groups (Fig. 6C). In addition, the percentage of MBP-positive cells was significantly increased in CM-ZJU37 (10 μM) group (Fig. 6E), which means that ZJU-37 promoted OPCs differentiation into mature oligodendrocytes in vitro. In accordance with observations from in vivo experiments, the results indicate that ZJU-37 suppressed apoptosis and promoted the survival and differentiation of OPCs.

## Discussion

White matter damage is a clinically important aspect of several CNS diseases in preterm infants, for which no specific treatments are available [6]. With improvements in obstetrics and intensive medical care, the condition has improved but approximately 35% of preterm infants suffer chronic neurological disorders [5]. Studies show that nervous system development in SD rats at postnatal 2–5 days is equivalent to that in human preterm infants at 23–32 weeks of gestation [42]. Current studies using neonatal animal WMI models have employed electrocoagulation of the right common carotid artery in P3 SD rats after hypoxia to discover new therapeutic strategies [43]. Recent evidence suggests that ZJU-37 plays an important role in cell death processes and inhibiting inflammatory responses but does not induce apoptosis. Here, we show for the first time that ZJU-37 not only suppresses NLRP3 inflammasome activation, attenuates OPC apoptosis and dysdifferentiation in vitro and in vivo but also promotes myelination and improves behavioral function in a neonatal rat WMI model with hOPC graft. Thus, this could be a feasible therapeutic approach to neonatal WMI.

Adverse factors in brain injury, such as infection and hypoxia, lead to OPCs damage and subsequent termination of the maturation process of oligodendrocytes, resulting in pathological changes including myelin dysplasia, decreased white matter volume, and ventricular enlargement in preterm infants [8]. Meanwhile, oligodendrocytes degeneration and necrosis and abnormal OPCs differentiation and maturation are major histological features. Stimulating the differentiation of OPCs into myelinating oligodendrocytes is a viable therapeutic option in WMI. Previous studies have reported [11, 14, 22] the differentiation of endogenous OPCs into myelin through drug therapies, which was previously tested in many animal models and clinical trials. For instance, Najm et al. [44] reported that miconazole and clobetasol promoted OPC differentiation and remyelination in an experimental autoimmune encephalomyelitis model. For genetic or congenital defects whose endogenous remyelination is unsuccessful, transplantation of exogenous cells is a promising treatment. Increasing evidence suggests that OPCs transplantation exerts its actions through reducing the loss of endogenous oligodendrocytes and stimulating endogenous progenitors proliferation [21]. Extensive animal model studies have demonstrated the feasibility and relative effectiveness of OPCs transplantation as reflected by increased remyelination [11, 38]. Currently, human cell sources for OPCs transplantation include human embryonic stem cells [20], human induced pluripotent stem cells [45], and NSCs [46]. However, the clinical application of these cells shows many limitations, such as the risk of tumorigenesis, the low survival rate of transplanted cells because of the lack of a more pro-survival milieu for OPCs, and high costs. Our previous studies indicated that although hOPCs transplantation helps to improve demyelination in mice with leukodystrophy, it cannot fully reverse the pathological status of damaged myelin [24]. Additionally, individual small-molecule functions have been revealed in OPCs [33] and ZJU-37 has been proved to effectively protect myelin sheath in animal models of several demyelinating diseases. However, it is unknown whether ZJU-37 exerts positive effects on hOPCs by preserving both structural and functional white matter integrity following neonatal WMI. To this end, upon the transplantation of hOPCs into the neonatal rat WMI model, ZJU-37 was injected intraperitoneally, and by 12 weeks, an increased MBP positive nerve fiber bundles was detected in the corpus callosum. In the present study, hOPCs effectively survived, migrated to the injured region and differentiated into mature oligodendrocytes expressing MBP in vivo with ZJU-37 treatment, which is exciting. Ultrastructural studies further confirmed the presence of new myelin sheaths. The g-ratio and score of myelin damage decreased after ZJU-37 combined with hOPC graft in the neonatal rat WMI model. ZJU-37 exerted a positive effect that exert remyelination function. Hypoxia and ischemia-induced OPCs progressively undergo apoptosis [5]. Our data show that the apoptosis OPCs increased in WMI rats, which is consistent with the results of previous reports. ZJU-37 combined with hOPCs more effectively decreased OPCs apoptosis compared to ZJU-37 or hOPCs treatment alone, as confirmed by PDGFRα immunofluorescent and TUNEL staining. One limitation of the present study was that we did not evaluate the differentiation of transplanted cells. Therefore, whether myelin repair occurred because of the transplanted cells or the differentiation of endogenous cells requires further analysis.

Previous research has shown that the neonatal rat WMI model display decreased motor function and impaired spatial working memory [11, 40]. In the present study, we provided in vivo evidence that ZJU-37 combined with hOPCs improved cognitive and motor function compared to ZJU-37 or hOPCs alone in WMI rats, as shown by behavioural tests. The ZJU-37 + hOPCs group showed greater improvement in the MWM test, cylinder test and adhesive removal test than the scramble group, suggesting that ZJU-37 facilitated cognitive functional improvement by decreasing OPCs apoptosis, and promoting OPCs differentiation and remyelination. However, the question remains: What is the mechanism underlying this recovery phenomenon?

The ischemia-hypoxia mediated OPCs apoptosis appears to be related to inflammation [41]. Increasing evidence has demonstrated that glial activation reactions are important self-protection mechanisms, which is a key factor in the promotion of functional recovery; however, the hyperactivation of glial cells is detrimental and induces demyelination by inhibiting OPC migration and differentiation, as well as inducing oligodendrocytes death [13, 41, 47]. Our previous studies demonstrated that astrocyte and microglia activation are consistently observed in animal models of demyelination and the inhibition of NLRP3 inflammasome activation alleviated cuprizone-induced demyelination [9, 25, 30]. This later study was particularly important, as it provided a foundation for the present study; Here, we found that ZJU-37 or/and hOPCs treatment significantly decreased the numbers of GFAP-positive and Iba-1-positive cells in the neonatal rat WMI model. Therefore, we explored how ZJU-37 protects the myelin sheath against inflammation by detecting the protein levels of NLRP3, ASC and cleaved-caspase-1 in the corpus callosum. Our data indicated that ZJU-37 inhibited glial and NLRP3 inflammasome activation and promoted OPCs survival and myelin regeneration in WMI rats with hOPC graft. This contributed to the functional integrity of white matter and recovery of neurological function and suggests that NLRP3 is involved in glial activation and neuroinflammation in WMI rats.

Astrocytes are highly secretory cells thought to be important in oligodendrogenesis following white matter damage [48]. Several studies have observed excessive astrocyte gliosis and inflammation in animal models of demyelination [9, 28]. Furthermore, apoptosis of OPCs is fundamental to the progression of demyelinating diseases, and astrocytes can induce OPCs death [48]. To investigate whether ZJU-37 decreased OPCs apoptosis induced by over-activation and inflammation of astrocytes, we used medium derived from OGD-injured astrocytes to treat OPCs. The results suggested that ZJU-37 suppressed NLRP3 inflammasome activation in OGD-induced astrocytes, and obviously attenuated OPC apoptosis and dysdifferentiation caused by the OGD-astrocyte-CM. This suggests that NLRP3 inflammasome activation in astrocytes triggers apoptosis in OPCs, which is critical for the pathogenesis of neonatal WMI and ZJU-37 suppressed this apoptosis of OPCs. Besides this, the immunofluorescence double staining showed ZJU-37 promoted OPCs differentiation into mature oligodendrocytes in vitro.

## Conclusions

In conclusion, the results demonstrated here show that the novel RIP1/RIP3 dual inhibitor ZJU-37 can effectively promote OPC survival and differentiation with hOPC graft, which alleviate hypoxia and ischemia-induced myelination and improves behavioural functions by targeting the NLRP3 inflammasome complex. Furthermore, these results support the potential therapeutic value by suggesting the positive neuroprotective effects of ZJU-37 combined with hOPCs transplantation for ischemia and hypoxia caused neonatal WMI. As both astrocyte and microglia have a crucial role in demyelination, and because ZJU-37 inhibited NLRP3 inflammasome activation of astrocyte and microglia in vivo, we only evaluated its effect on astrocyte and not its direct effect on microglia. This should be addressed in further studies.

## Data Availability

The data that support the findings of this study are available from the corresponding authors upon reasonable request.

## Abbreviations

A2B5: ST8 alpha-*N*-acetyl-neuraminide alpha-2,8-sialyltransferase 1
ASC: Apoptosis associated speck-like protein containing caspase recruitment domain
ANOVA: Analysis of variance
BBB: Blood–brain barrier
CM: Conditioned medium
CNS: Central nervous system
DAPI: 4′,6-Diamidino-2-phenylindole
ESCs: Embryonic stem cells
FBS: Fetal bovine serum
GFAP: Glial fibrillary acidic protein
hOPCs: Oligodendrocyte precursor cells derived from human neural stem cells
Iba-1: Ionized calcium binding adaptor molecule-1
MBP: Myelin basic protein
MWM: Morris water maze
NG2: Chondroitin sulfate proteoglycan 4
NLRP3: Nod-like receptor pyrin domain containing 3
NSCs: Neural stem cells
OGD: Oxygen–glucose deprivation
OGD-astrocyte-CM: The conditioned medium from OGD-injured astrocytes
OPCs: Oligodendrocyte precursor cells
PBS: Phosphate buffer saline
PDGFRα: Platelet derived growth factor receptor alpha
pre-OLs: Pre-oligodendrocytes
PRRs: Pattern recognition receptors
RIP1/RIP3: Receptor interacting protein kinase-1/-3
SD: Sprague-Dawley
TUNEL: Terminal deoxynucleotidyl transferase dUTP nick end labeling
WMI: White matter injury
